# A new approach for analyzing an adhesive bacterial protein in the mouse gastrointestinal tract using optical tissue clearing

**DOI:** 10.1038/s41598-019-41151-y

**Published:** 2019-03-18

**Authors:** Keita Nishiyama, Makoto Sugiyama, Hiroki Yamada, Kyoko Makino, Sayaka Ishihara, Takashi Takaki, Takao Mukai, Nobuhiko Okada

**Affiliations:** 1 Department of Microbiology, School of Pharmacy, Kitasato University, Minato-ku, Tokyo, 108-8641 Japan; 2 Faculty of Veterinary Medicine, School of Veterinary Medicine, Kitasato University, Towada, Aomori, 034-8628 Japan; 3 Department of Biosciences, School of Science, Kitasato University, Sagamihara, Kanagawa 252-0373 Japan; 4 Section of Electron Microscopy, Showa University, Shinagawa-ku, Tokyo, 142-8555 Japan; 5 Department of Animal Science, School of Veterinary Medicine, Kitasato University, Towada, Aomori, 034-8628 Japan

## Abstract

Several bacterial moonlighting proteins act as adhesion factors, which are important for bacterial colonization of the gastrointestinal (GI) tract. However, little is known about the adherence properties of moonlighting proteins in the GI tract. Here, we describe a new approach for visualizing the localization of moonlighting protein-coated fluorescent microbeads in the whole GI tract by using a tissue optical clearing method, using elongation factor Tu (EF-Tu) as an example. As a bacterial cell surface-localized protein mimic, recombinant EF-Tu from *Lactobacillus reuteri* was immobilized on microbeads. EF-Tu-coating promoted the interaction of the microbeads with a Caco-2 cell monolayer. Next, the microbeads were orally administered to mice. GI whole tissues were cleared in aqueous fructose solutions of increasing concentrations. At 1 h after administration, the microbeads were diffused from the stomach up to the cecum, and after 3 h, they were diffused throughout the intestinal tract. In the lower digestive tract, EF-Tu-beads were significantly more abundant than non-coated control beads, suggesting that EF-Tu plays an important role in the persistence of the microbeads in the GI tract. The new approach will help in evaluating how moonlighting proteins mediate bacterial colonization.

## Introduction

Moonlighting refers to the ability of proteins or peptides to exert multiple biologically important functions in plants, animals, yeasts, and bacterial organisms^1–3^. Moonlighting proteins are well characterized in bacteria: in their cytoplasmic form, these proteins have canonical functions in essential cellular processes, such as glycolysis, chaperone activity, protein synthesis, and nucleic acid stability, whereas in their cytoplasmic form, following secretion and localization to the cell surface, they often exert additional functions (i.e., “moonlight”) as cell-surface proteins^3–5^. In gut microbes, cell-surface components are the first point of contact with the host mucosal surface. The most commonly identified moonlighting function of these cytoplasmic proteins is adhesion. The first bacterial adhesive moonlighting protein to be identified was glyceraldehyde-3-phosphate dehydrogenase (GAPDH), isolated from the cell surface of *Streptococcus pyogenes*, which may be involved in colonization and internalization^6^. Later, several cytoplasmic proteins were found to have a moonlighting function in adhesion to epithelial cells, extracellular matrix proteins, glycocalyx, and mucins in pathogenic and commensal bacteria^4–7^.

The localization of moonlighting proteins on the cell surface has been suggested based on their ionic interactions^8–10^. They are released from cells and bind to neighboring bacterial cells^5^. Therefore, it is difficult to determine which target protein is derived from which cell. In addition, as most moonlighting proteins are essential for bacterial growth, gene inactivation is lethal. These distinctive properties have hampered the characterization of adhesive bacterial moonlighting proteins, especially *in vivo*.

Some studies reported that genetic modification of specific moonlighting proteins, including GAPDH from *Streptococcus pyogenes* and enolase from *Bacillus subtilis*, prevented their secretion^11,12^. Genetic fusion of the hydrophobic tail at the C terminus abolished the cell surface translocation of GAPDH in *S. pyogenes*^11^. Using this mutant, the authors showed that GAPDH is involved in *S. pyogenes* adherence and antiphagocytic properties^11^ and thus, is essential for *S. pyogenes* virulence^13^. However, these research have been obtained only for enolase and GAPDH.

Carboxylate-modified microbeads can be modified with several biomolecules, such as antibodies and adhesion factors, which can interact with the cells of a wide array of organisms and thus confer biological functionality to the microbeads. For example, beads modified with CD47, a member of the immunoglobulin superfamily, inhibit tumor development in mice and have shown promise in immunotherapeutic approaches^14^, and beads modified with multivalent adhesion molecules prevent *Pseudomonas aeruginosa* infection in mice^15^. Based on these reports, we hypothesized that microbeads coated with a single moonlighting protein could be useful for evaluating its moonlighting function (in this case, as an intestinal adhesion factor).

Optical tissue clearing has been used as a powerful tool for deep imaging of mammalian tissues, such as neuronal networks in the whole mouse brain^16,17^. Ke *et al*.^18^ established SeeDB (See Deep Brain), which uses a high-refractive-index aqueous optical clearing agent composed of fructose and thiol to detect fluorescence signals in whole tissues. The elongation factor Tu (EF-Tu), which is one of the most abundant proteins in bacteria involved in protein synthesis^19^, has been shown to display moonlighting activities, such as multiple adhesive characteristics^10,20–24^. We previously found that the cell surface-associated EF-Tu from *Lactobacillus reuteri* binds to sulfated carbohydrate moieties of glycocalyx and mucins, suggesting that this protein is an adhesion factor in *L. reuteri*^10^.

In this study, we visualized the localization of recombinant EF-Tu-coated fluorescent microbeads in whole intestinal tissue using the tissue optical clearing method. The method reported here is a new approach to visualizing the behavior of moonlighting protein-functionalized fluorescent microbeads in the whole gastrointestinal (GI) tract of mice.

## Results

### Immobilization of recombinant EF-Tu proteins onto carboxylate microbeads

**Figure 1:**
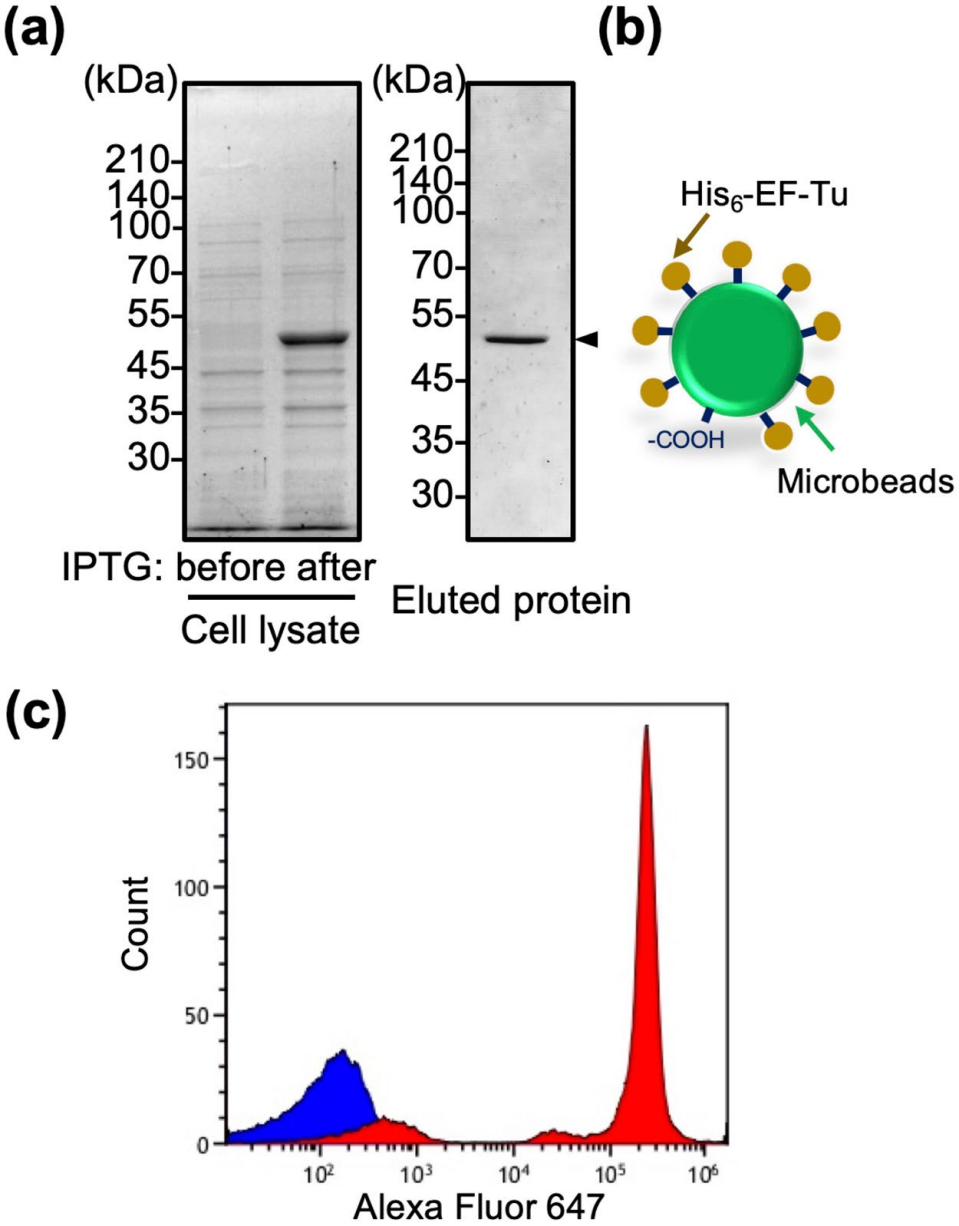
Immobilization of recombinant EF-Tu protein onto carboxylate microbeads. (**a**) Expression and purification of EF-Tu protein from *L. reuteri* JCM1112^T^. Whole cell lysate or nickel-purified fractions derived from *E. coli* expressing His_6_-EF-Tu (indicated by arrow) were resolved by sodium dodecyl sulfate-polyacrylamide gel electrophoresis and stained with Coomassie blue. Full-length gels are presented in Supplementary Fig. S6. (**b**) Schematic representation of His_6_-EF-Tu bead mimicry of *L. reuteri* EF-Tu. Carboxy-modified polystyrene microbeads of 1.5 μm in diameter were activated using EDC/NHS, and covalently coupled with His_6_-EF-Tu. (**c**) Flow-cytometric measurement of His_6_-EF-Tu immobilized on microbeads (red area) in comparison with non-coated control beads (blue area).

EF-Tu from *L. reuteri* JCM1112^T^ was expressed as a His_6_-tagged protein in *E. coli*. An approximately 47-kDa band was clearly observed following induction with IPTG (Fig. 1a). The crude extract was purified as described in the Materials and Methods section. Expression and purification of His_6_-EF-Tu was verified by sodium dodecyl sulfate-polyacrylamide gel electrophoresis followed by staining with Coomassie brilliant blue (Fig. 1a, eluted protein). The purified recombinant EF-Tu was chemically coupled to microbeads, and the complex mimicked a bacterial cell surface-localized protein (Fig. 1b). Immobilization of His_6_-EF-Tu on the microbeads was confirmed by flow cytometry using an anti-EF-Tu antibody. As shown in Fig. 1c, strong fluorescence signal was detected for recombinant EF-Tu-beads, but not for control-beads. Thus, EF-Tu-beads were successfully generated.

### Recombinant EF-Tu on microbeads promotes protein-host cell interaction

**Figure 2:**
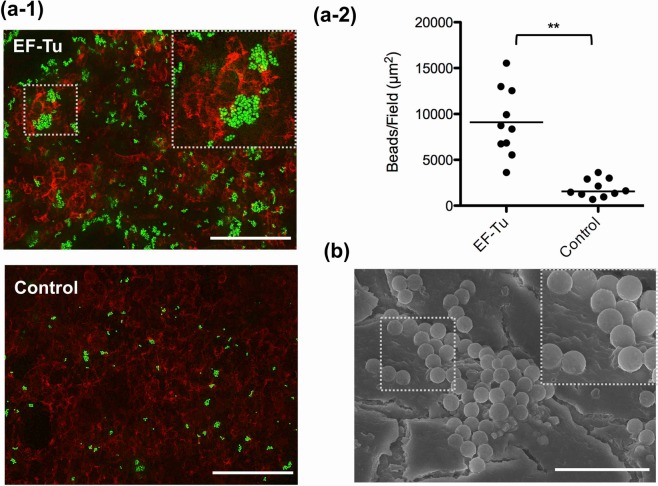
Recombinant EF-Tu-functionalized fluorescent microbeads promote beads-Caco-2 cell interaction. (**a-1**) Fluorescence microscopic image of microbeads adherent to Caco-2 cells. EF-Tu-beads and non-coated control beads (green) were added to Caco-2 cell monolayers and incubated for 1 h. For counter staining, cells were labeled for F-actin (red). Scale bars, 100 μm. (**a-2**) Bound beads were quantified using a Pulse-SIM BZ-X700 microscope equipped with the Hybrid Cell Count BZ-H3C software. Each dot indicates the average fluorescent area (μm^2^) in five randomly selected fields. Adhesion test was repeated individually 10 times. Bars indicate median. **P < 0.01, EF-Tu-beads *vs*. control-beads (one-tailed Mann–Whitney U test). (**b**) SEM images of microbeads adhered to Caco-2 cells. Scale bars. 5 μm.

A previous study indicated that *L*. *johnsonii* NCC533 (La1) EF-Tu binds to Caco-2 cells^20^. To examine whether the recombinant EF-Tu was able to promote the adhesion of microbeads to Caco-2 cells, we visualized the localization of the microbeads on cell monolayers using fluorescence microscopy. When Caco-2 cells were treated with EF-Tu-beads or control-beads, EF-Tu-beads markedly localized on the cell monolayer (Fig. 2a-1) and directly interacted with the cell surfaces (Fig. 2b). The area of bead adherence was significantly (*P* < 0.01) higher for EF-Tu- than for control-beads (Fig. 2a-2). Bovine serum albumin (BSA)-beads adhered to Caco-2 cells at a level similar to that observed for control beads (Supplementary Fig. S1a,b). This result indicated that the recombinant EF-Tu promotes the interaction between microbeads and Caco-2 cells.

### Visualization and quantification of microbeads in mouse whole GI tissues using optical tissue clearing

**Figure 3:**
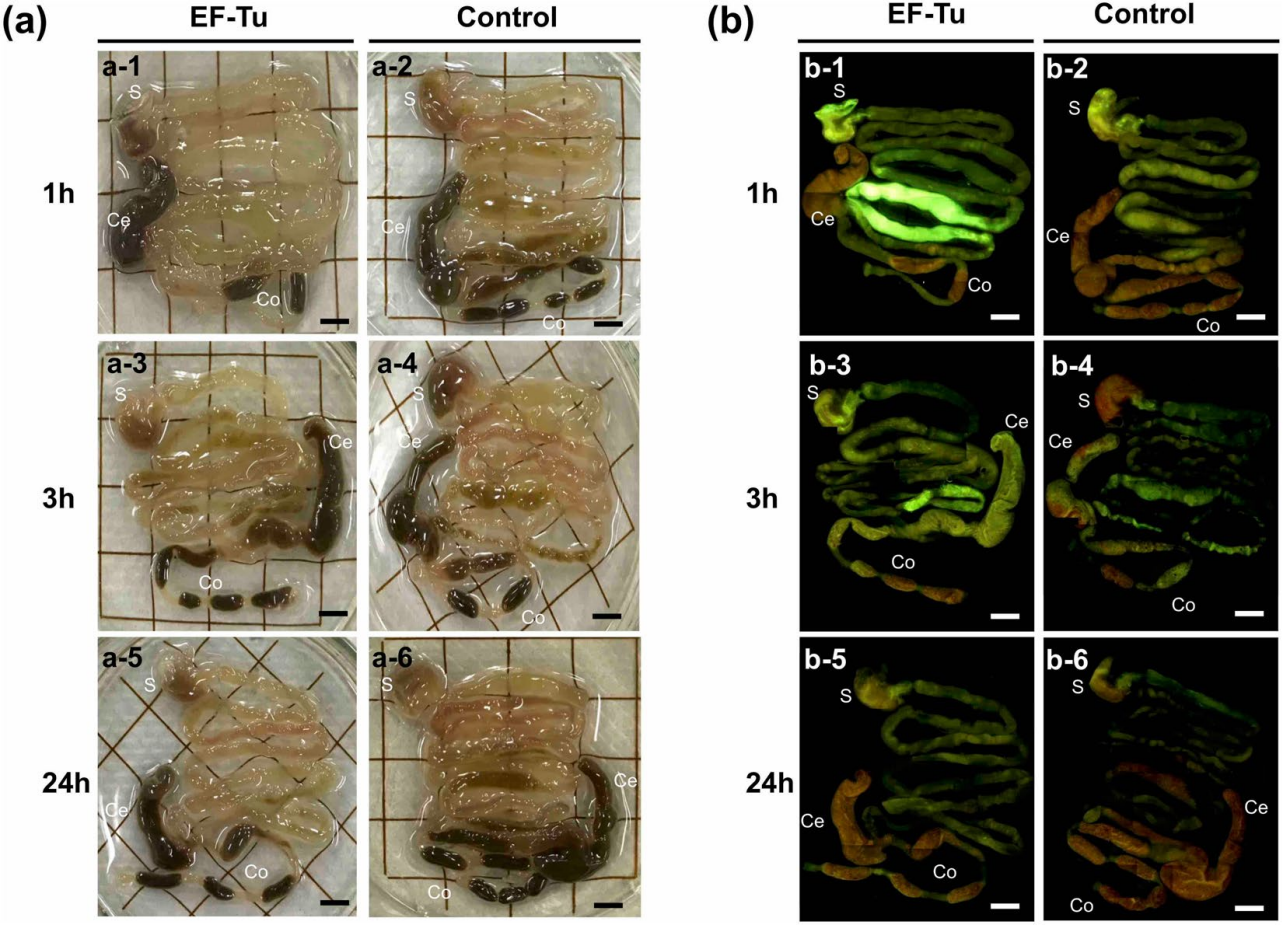
Visualization of the localization of recombinant EF-Tu-functionalized fluorescent microbeads in mouse whole GI tract using the tissue clearing method. EF-Tu-beads or control-beads were orally administered to mice. At 1, 3, and 24 h after administration, whole GI tissues were treated with increasing concentrations of aqueous fructose solutions for tissue clearing. Whole-gut images were acquired using a Leica M205 fluorescence stereomicroscope under (**a**) bright light or (**b**) fluorescence. All data are representative of two independent experiments. Identical symbols indicate the same mouse tissue. Scale bar, 5 mm. S, stomach; Ce, cecum; Co, colon.

**Figure 4:**
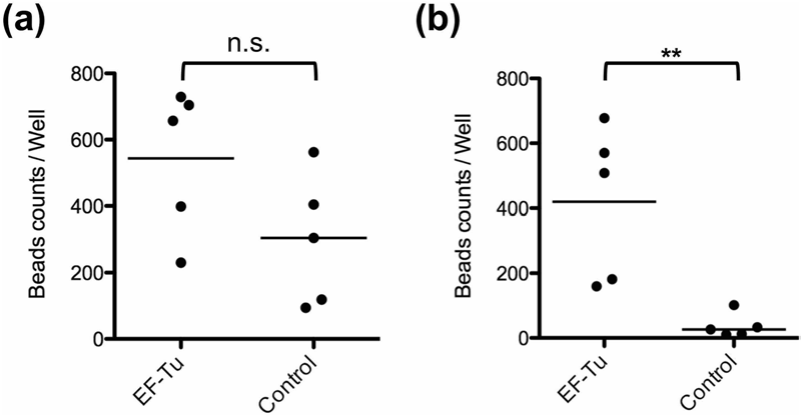
EF-Tu impacts the persistence of microbeads in the mouse GI tract. Quantification of EF-Tu-beads and control-beads in the mouse GI tract at 24 h after administration. Tissues of the (**a**) upper digestive tract (duodenum to ileum) and (**b**) lower digestive tract (cecum to colon) were separated. Total intestinal contents were collected and evaluated on a diagnostic glass slide (15 wells per sample) (see Materials and methods). Microbeads in each well were counted by fluorescence microscopy using a Pulse-SIM BZ-X800 microscope equipped with the Hybrid Cell Count BZ-H4C software. Each plot indicates average number of beads per intestinal tissue sample (15 wells; n = 5 mice per group). Bars indicate the median. n.s., non-significant difference, **P < 0.01, EF-Tu-beads vs. control-beads (one-tailed Mann–Whitney U test).

To confirm whether the recombinant EF-Tu coating affected the persistence of the microbeads in the mouse GI tract, microbeads were orally administered to mice. At 1, 3, and 24 h after administration, the mice were sacrificed. Both groups of mice displayed similar appetites during the experiment. The GI tissues were harvested and treated with increasing concentrations of aqueous fructose solutions, and then equilibrated in saturated fructose solution (Supplementary Fig. S2a,b). Tissue clearance was achieved without loss of optical clarity (Supplementary Fig. S2a), and intrinsic fluorescence was not detected (Supplementary Fig. S2c). Images of whole GI tissues cleared using optical clearing agent are provided in Fig. 3a,b. At 1 h after administration, the microbeads were diffused throughout the stomach up to the cecum (Fig. 3b-1 and -2). Strong fluorescence signal was observed around the ileum in the mice treated with the EF-Tu-beads (Fig. 3b-1). At 3 h after administration, the microbeads reached the colon and were diffused throughout the intestinal tract (Fig. 3b-3 and -4). The fluorescent signal of control-beads was weaker than that of EF-Tu-beads. Fluorescent signal of both types of beads was observed throughout the GI tract even at 24 h after administration (Fig. 3b-5 and -6). Furthermore, the localization pattern of fluorescence microbeads differed between the upper digestive tract (duodenum to ileum) and the lower GI tract (cecum to colon) (Fig. 3b-5 and -6). When beads coated with wheat germ agglutinin (WGA), which binds to mucin carbohydrates^25^, were administered, enhanced fluorescence was observed around the jejunum (Supplementary Fig. S3b-1), which was notably different when compared with that observed in BSA- and control-beads at 3 h after administration (Supplementary Fig. S3b-2 and -3). These results indicated that a protein immobilized on the fluorescent microbeads affects the localization of microbeads *in vivo*.

Microbeads in the upper and lower tract were quantified by counting under a fluorescence microscope at 24 h after administration (Fig. 4). The number of microbeads in the upper digestive tract was not significantly different between mice administered EF-Tu-beads and those administered control-beads (Fig. 4a). In contrast, in the lower digestive tract, the number of EF-Tu-beads was significantly higher (approximately 19-fold) than that of control-beads (Fig. 4b). Micro-imaging of the colon tissues revealed that EF-Tu-beads were localized at the mucosal surface (Supplementary Fig. S4, arrows). In addition, histological analysis of distinct intestinal regions in mice by high iron diamine-alcian blue staining indicated that the number of sulfomucin-containing goblet cells, which are a receptor for EF-Tu^10^, gradually increased in the lower intestine (Supplementary Fig. S5). Collectively, our results demonstrate that EF-Tu plays an important role in bacterial colonization of the GI tract, especially, the lower digestive tract.

## Discussion

The colonization characteristics of commensal or pathogenic bacteria in the GI tract are important to understand their potential survival strategies. Several bacterial moonlighting proteins function in adhesion, which has been considered the key process in bacterial colonization of the host mucosal surface^3–5^. Here, we describe a new approach for visualizing the time-course behavior of moonlighting protein-functionalized fluorescent microbeads as a model of the protein-bacterial complex in the mouse whole GI tract by optical tissue clearing, using EF-Tu as an example.

Bioluminescent signal-based *in-vivo* imaging was first reported in mice infected with *lux*-tagged *Salmonella* Typhimurium^26^. Subsequently, luminescent imaging was widely reported in various pathogens^27–30^ and commensal bacteria, such as *Bifidobacterium* and *Lactobacillus*^31–33^. This method can be used to evaluate how colonization factors contribute to the GI transit time of bacteria as well as their spatial localization^29,30^. However, genetic manipulation is required to introduce marker genes, and mutations must be made in genes encoding adhesion factors. In contrast, our approach is simple and can be used to evaluate the functions of moonlighting proteins, which are adhesive in nature, using *E. coli*-expressed recombinant proteins immobilized on microbeads to mimic bacterial cell-surface proteins. Most moonlighting proteins are cytoplasmic proteins, and recombinant proteins expressed in the cytoplasm of *E. coli* are more soluble than membrane proteins^34^. Therefore, *E. coli*-expressed recombinant proteins have been used to evaluate several moonlighting proteins as adhesion factors, such as EF-Tu^10,20^, enolase^35^, GAPDH^36^, streptococcal surface dehydrogenase SDH^37^, glutamine synthetase^38^, and glucose-6-phosphate isomerase^38^. Therefore, we expect that various moonlighting proteins can be examined using our system.

We visualized the localization of fluorescent microbeads in whole GI tract using tissue optical clearing. Various water-based tissue clearing agents, such as SeeDB and Scale, which are used to visualize spatial neuronal networks in the brain, have been reported^16,18,39^. Compared to the Scale method^16^, SeeDB is faster and minimizes morphological tissue distortion^18^. However, these protocols are optimized for studying neuronal connectivity and imaging tissues deep inside the brain. To enable clearing of the whole GI tract, which is thin and tubular in shape, without loss of mucus and to trace fluorescence microbeads in the murine GI tract, we optimized the clearing process. We modified the SeeDB clearing protocol as follows: (i) some immersion times and steps were shortened, and the entire process was completed within 36 h; (ii) to allow the clearing solution to quickly permeate, tissues were soaked in a low concentration of surfactant during the washing steps. Although the samples treated with the modified protocol were moderately cleared as compared to when the original SeeDB method was used, the tissue was sufficiently cleared to detect the fluorescent microbeads in the murine GI tract. This optimized clearing protocol prevented tissue deformation artifacts and reduced the overall cost as lower of amounts of reagents were used. Moreover, intact tissues spread on a dish were imaged using an optical microscope, obliterating the need for specialized equipment, such as a confocal or two-photon microscope.

Interestingly, the microbeads were diffused throughout the murine GI tract, from stomach to colon, but the localization and density of the beads clearly varied between different sites in the GI tract (Fig. 3). Particularly, regardless of EF-Tu-coating, the fluorescent signal was stronger in the upper than in the lower digestive tract. According to previous research, the small intestine has loose and penetrable mucus, which allows easy penetration of microbeads (0.5–2.0 μm in diameter). In contrast, the mucus layer of the distal colon is not penetrable to beads, and that of the proximal colon is only partly penetrable^40^. The small intestinal mucus may allow easy penetration of nutrients, in contrast to the colon, where the mucus physically protects the epithelial cells from bacteria and feces. In fact, the penetration of microbeads in mucus is related to their retention in the GI tract^41^. We reason that these physiological differences in mucus composition may have affected the spatial localization of microbeads in the GI tract in the current study.

Whereas EF-Tu-beads were significantly more abundant than control-beads in the lower digestive tract, their numbers were not different in the upper digestive tract. We previously reported that recombinant EF-Tu from *L. reuteri* JCM1081 binds to sulfated carbohydrate moieties of glycocalyx and mucins^10^. Moreover, sulfomucin-containing goblet cells are substantially more abundant in the lower than in the upper digestive tract (Supplementary Fig. S5). Based on these facts, we predicted that microbead-bound EF-Tu would be retained longer through specific binding ability in the lower digestive tract. As WGA-beads also exhibited a site-specific localization *in vivo*, the adhesion factor-functionalized fluorescent microbeads might reflect the specific colonization site of a bacterium in the GI tract.

However, micro-imaging of colon tissues revealed that EF-Tu-beads were aggregated on the mucosal surface. For intestinal bacteria, cell-surface protein-mediated bacterial aggregation has been considered to provide a colonization advantage^42–44^. Actually, since non-adhesion BSA-beads also persisted in the GI tract compared with control-beads, the spatial localization of the microbeads may depend on the immobilized protein and may involve a complex process including hydrophobic and/or electrical forces. EF-Tu-mediated host adhesion and/or aggregation might be advantageous in the persistence of the intestinal bacteria (mimicked by the microbeads), especially in the lower GI tract. Therefore, we believe that the visualization of individual moonlighting proteins is an attractive approach for evaluating their functions in the whole GI tract.

There are several limitations to analyzing moonlighting protein-functionalized fluorescent microbeads in the whole GI tract of mice. First, we used the microbeads as a size mimic of bacteria, which does not allow considering effects of factors such as stress resistance and growth rate on bacterial survival. Second, we did not evaluate protein steric hindrance induced by immobilization. Third, to comprehensively analyze the importance of EF-Tu as a bacterial adhesion protein *in vivo*, further comparative analyses using other adhesion factors, including adhesive moonlighting proteins, using this system need to be performed.

In conclusion, we established a new experimental system for evaluating the behavior of moonlighting proteins *in vivo*, which is generally difficult, by using putative moonlighting protein-functionalized fluorescent microbeads and tissue clearing techniques. We expect that our approach will be widely used to evaluate the mechanisms by which moonlighting proteins promote bacterial colonization.

## Materials and Methods

### Animals

Eight-week-old C57BL/6JJcl male mice were purchased from CLEA Japan, Inc. (Tokyo, Japan). All mice were housed in SPF conditions at 22 ± 2 °C under a 12-h:12-h light/dark cycle with *ad libitum* access to food and water. The animals were acclimated for one week prior to dosing. The mice were administered microbeads at nine weeks of age. Animal care and experiments were approved by the President of Kitasato University through the Institutional Animal Care and Use Committee of Kitasato University (approval no. 18-040), and all animal experiments were conducted in accordance with the approved guidelines.

### Epithelial cell line and culture conditions

The epithelial cell line Caco-2 was obtained from the RIKEN Cell Bank (Tsukuba, Japan). The cells were cultured in Dulbecco’s modified Eagle’s medium (Sigma-Aldrich, St. Louis, Missouri) supplemented with 10% (v/v) fetal bovine serum, 10 U/mL penicillin, and 10 μg/mL streptomycin. The cells were maintained in 25-cm^2^ flasks and then seeded onto eight-chamber glass slides (Fukaekasei and Watson, Hyogo, Japan). The cells were then grown to confluence for 10 days at 37 °C in the presence of 5% CO_2_, and the medium was changed every two days.

### Expression and purification of recombinant proteins in *Escherichia coli*

Recombinant protein was expressed in *E. coli* and purified with a His_6_-tag at the N-terminus. *L. reuteri* JCM1112^T^ was obtained from the Japan Collection of Microorganisms, RIKEN BioResource Research Center (Tsukuba, Japan). The *L. reuteri* JCM1112^T^ *tuf* gene, which encodes EF-Tu (accession no. BAG25144.1) was amplified by PCR using Ex taq DNA Polymerase (Takara Bio Inc., Shiga, Japan) and the primer pair 5′-CATATGGCTGAAAAAGAACATTATGAAC-3′ and 5′-CTCGAGTTAGTCTAAGATGTCGGATAC-3′. The amplified fragments were inserted into the pET28b vector (Novagen, WI, USA) containing NdeI and XhoI restriction sites. Successful insertion was confirmed by sequencing, and the plasmid was used to transform *E. coli* Rosetta 2 (DE3).

Transformed cells were grown in Luria-Bertani medium at 37 °C with shaking at 160 rpm. When the OD_600_ reached approximately 0.4, the cells were incubated with isopropyl-β-d-thiogalactopyranoside (0.3 mM) for 4 h to induce protein expression. Then, the cells were lysed using BugBuster Protein Extraction Reagent (Novagen), 0.2 mg/ml lysozyme (Sigma-Aldrich), 20 μg/ml DNase (Sigma-Aldrich), 1 mM MgCl_2_, and 1 mM phenylmethylsulfonyl fluoride (Sigma-Aldrich). The cellular debris was removed by centrifugation (16,000 × *g*, 10 min, 4 °C), and the supernatant was passed through a syringe filter (0.22 μm pore size). His_6_-tagged EF-Tu was purified using a HisTrap HP column (1 mL; GE Healthcare) in line with the ÄKTA start (GE Healthcare) according to the standard operating procedure. Purified proteins were dialyzed in 20 mM HEPES buffer (pH 7.0). Protein concentrations were determined using a Bicinchoninic Acid Protein Assay kit (Takara Bio Inc.).

### Immobilization of recombinant EF-Tu protein on microbeads

Recombinant EF-Tu protein was coupled with Fluoresbrite^®^ YG Carboxylate microbeads (Ф1.5 μm [Polysciences, Inc., Warrington, PA]) using the PolyLink Protein Coupling Kit (Polysciences, Inc.) following the manufacturer’s instructions. Microbeads (12.5 mg) were centrifuged for 10 min at 1,000 × *g* at 25 °C. The pellet was resuspended in 400 μL of PolyLink coupling buffer and centrifuged for 10 min at 1,000 × *g*. The beads were then resuspended in 170 μL of PolyLink coupling buffer. To activate the beads for protein coupling, 20 μL of EDAC solution (200 mg/mL) was added and mixed gently. To this solution, 100 μg of purified EF-Tu protein (His_6_-tagged EF-Tu protein) was added, and the mixture was incubated for 60 min at room temperature with gentle shaking. The beads are washed twice by centrifugation with PolyLink wash/storage buffer for 10 min at 1,000 × *g*. The pellet was re-suspended in 400 μL of 50 mM phosphate buffer (pH 7.5) containing 150 mM NaCl and 0.05% (w/v) bovine serum albumin and stored at 4 °C until use. Immobilization of BSA (Sigma-Aldrich) and WGA (J-chemical co. ltd, Tokyo, Japan) was conducted using the same procedure. Non-coated beads were only activated, without protein coupling.

Coupling of EF-Tu with the beads was confirmed by flow cytometry. The beads were mixed with anti-EF-Tu polyclonal antibody^10^ (1:100 dilution) and incubated for 45 min. Then, the beads were washed twice with PBS containing 0.01% (w/v) Tween-20 and incubated with anti-rabbit IgG H&L-Alexa Fluor 647 secondary antibody (ab150079, Abcam) for 45 min in dark. Control-microbeads stained with primary and secondary antibodies were used as a control. Flow cytometry was performed using a CytoFLEX flow cytometer (Beckman Coulter) according to the manufacturer’s instructions.

### *In-vitro* adhesion test

Caco-2 cells were cultured in 8-chamber slides and fixed with 4% paraformaldehyde at room temperature for 5 min. Then, 50 μL of microbeads was mixed with 450 μL of PBS (i.e., 10-fold dilution). The suspension (100 μL) was added to each well and incubated at room temperature for 1 h. After washing three times with 100 μL of PBS, the cells were stained with Acti-stain 555 phalloidin (PHDH1-A; Cytoskeleton, Inc., Denver, CO, USA) to visualize actin filaments. Fluorescence images were obtained using a Pulse-SIM BZ-X700 microscope equipped with the Hybrid Cell Count BZ-H3C software (Keyence, Osaka, Japan).

For scanning electron microscopy (SEM), the slide was mounted on aluminum stubs with double-sided carbon tape (Nisshin EM, Tokyo, Japan) and coated with platinum-palladium using E-1030 Ion sputter (Hitachi, Tokyo, Japan). Images were acquired using a Hitachi S-4700 FE-SEM (accelerating voltage, 10 kV).

### *In-vivo* colonization test

Before treatment, mice were fasted for 8 h. After gentle stirring, 100 μL of microbeads (approximately 5 × 10^6^ particles) was periorally administered to the mice. After treatment, the mice were allowed *ad libitum* access to food and water. The intestinal tissues were collected at 1, 3, and 24 h after administration.

Tissue clearing was carried out as described previously for the water-based optical clearing agent, SeeDB^18^, with several modifications to optimize the method for GI tissues. After dissecting the pancreas and mesentery, mice GI tissues were arranged in a sheet shape and were fixed in 4% paraformaldehyde at 4 °C overnight. Then, the tissues were treated with 0.1% Triton X-100 (v/v) in PBS for 30 min at room temperature, washed with PBS twice, and immersed successively in 20%, 40%, 80%, and 100% d(–) fructose (FUJIFILM Wako Chemicals, Osaka, Japan) solution (w/v) for 4, 4, 4, and 12 h, respectively, and the saturated solution was incubated for another 12 h. Each clearing step required approximately 100 mL of tissue clearing reagent. All clearing reagents were prepared in distilled water containing 0.5% (v/v) α-thioglycerol (Wako) and 0.01% (w/v) sodium azide. All steps were performed at room temperature under mild shaking. Whole-gut images were acquired with a fluorescence stereomicroscope (M205; Leica, Germany) using a 395–455 nm mercury short arc lamp. A series of images were aligned to construct a single image using Photoshop CC 2017 (Adobe Systems, CA, USA).

To count microbeads, intestinal tissues were separated into upper (duodenum to ileum) and lower (cecum to rectum) parts. These tissues were washed with 5 mL of PBS to obtain total intestinal contents. To the collected sample solutions, 1% (v/v) Triton X-100 was added and the mixture was incubated for 30 min at room temperature with shaking at 100 rpm. To remove large contents, the samples were centrifuged at 500 × *g* for 5 min. Then, 5 μL of the supernatant was dropped on a diagnostic glass slide (Ф 6 mm) (Matsunami Glass, Osaka, Japan). After drying, the total number of microbeads in each well (15 wells per sample) was counted under a Pulse-SIM BZ-X800 microscope equipped with the Hybrid Cell Count BZ-H4C software (Keyence).

### Statistical analysis

Means were compared using a one-tailed Mann–Whitney U test in Prism 6 (GraphPad Software). *p*-Values < 0.05 were considered statistically significant.

## Supplementary information

Supplementary Information
