# Supplementary material for: A new approach for analyzing an adhesive bacterial protein in the mouse gastrointestinal tract using optical tissue clearing
Source: Sci Rep. 2019 Mar 18;9:4731. doi: 10.1038/s41598-019-41151-y (PMC6426832; doi:10.1038/s41598-019-41151-y)
Supplement: Supplementary file 1 — Supplementary Information [file 41598_2019_41151_MOESM1_ESM.pdf]

Supplementary Information

A new approach for analyzing an adhesive bacterial protein in the mouse gastrointestinal tract using optical tissue clearing

Keita Nishiyama, Makoto Sugiyama, Hiroki Yamada, Kyoko Makino, Sayaka Ishihara, Takashi Takaki, Takao Mukai, Nobuhiko Okada

Supplementary Materials and Methods

Evaluation of the transmittance during optical clearing

Transmittance of small and large intestinal tissues during optical clearing was measured using ImageJ 1.49v. (NIH, Bethesda, MD, USA). Tissues were imaged at each step with a stereomicroscope under the same transmitted-light setup (Supplementary Fig. S2a). The images were converted to grayscale images, and then, the gray-color density was measured as the ratio of visible light transmission to the grid lines. The average of five values was normalized to "small intestine at 0 h" and these values were defined as tissue transmittance.

Histochemistry

Intestinal tissues of adult C57BL/6JJcl mice were fixed without washing in methanol-Carnoy's fixative at room temperature for 2 h[1]. Paraffin-embedded sections were dewaxed, hydrated, and cut into 4-μm sections. Histochemical detection of whole sulfated and sialylated glycoconjugates was performed by high iron diamine-alcian blue staining (pH 2.5)[2]. Images were acquired with an Olympus microscope (Tokyo, Japan).

Supplementary references

1. Nishiyama, K., Sugiyama, M. & Mukai, T. Adhesion properties of lactic acid bacteria on intestinal mucin. *Microorganisms* **4,** E34 (2016).
2. Nishiyama, K. *et al.* Identification and characterization of sulfated carbohydrate-binding protein from *Lactobacillus reuteri*. *PLoS One* **8,** e83703 (2013).

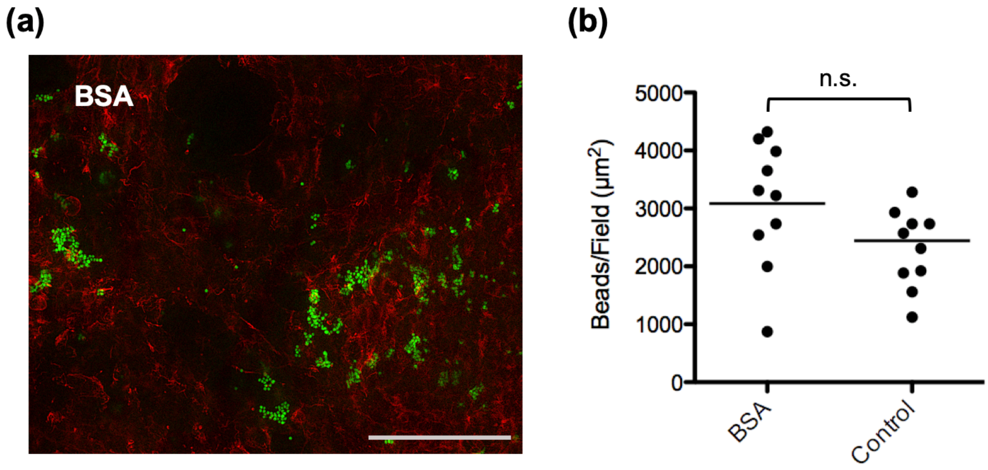

Figure S1. Bovine serum albumin (BSA)-functionalized fluorescent microbeads promote beads-Caco-2 cell interaction. (a) Fluorescence microscopic image of microbeads adherent to Caco-2 cells. BSA-beads and non-coated control beads (green) were added to Caco-2 cell monolayers and incubated for 1 h. For counter staining, cells were labeled for F-actin (red). Scale bars, 100 µm. (b) Bound beads were quantified using a Pulse-SIM BZ-X700 microscope equipped with the Hybrid Cell Count BZ-H3C software. Each dot indicates the average fluorescent area (µm^2) in five randomly selected fields. Adhesion test was repeated individually 10 times. Bars indicate median. n.s. indicating non-significant difference, BSA-beads *vs*. control-beads (one-tailed Mann–Whitney U test).

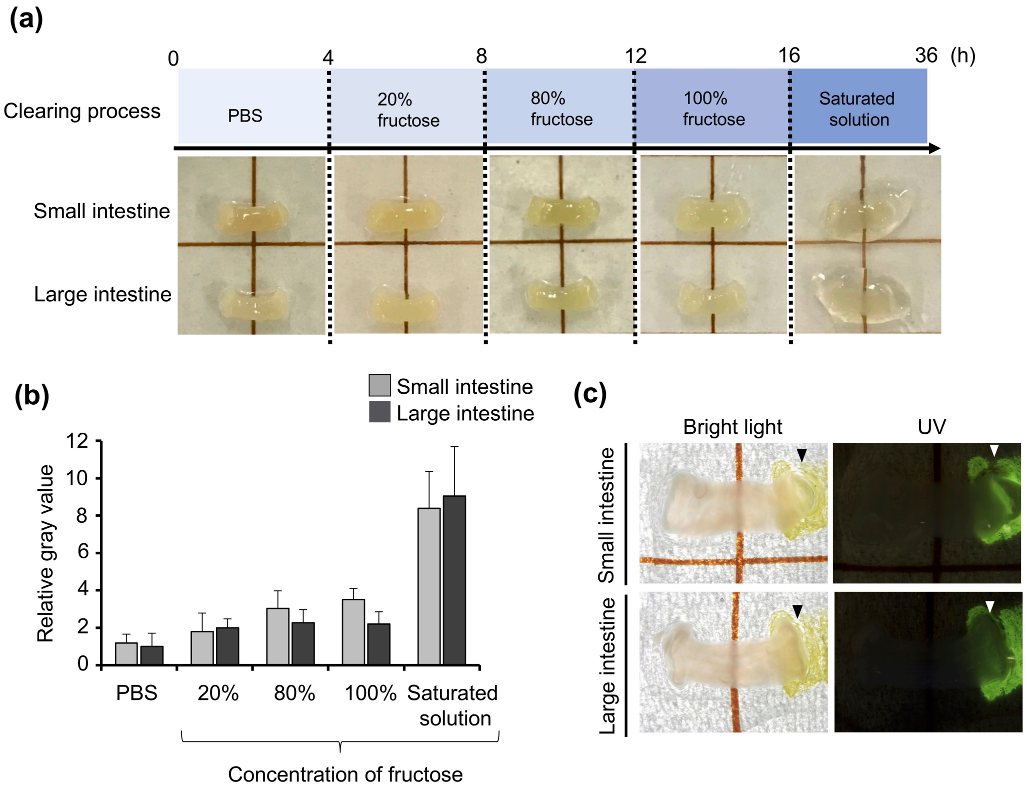

Figure S2. Schematic diagram of intestinal tissue clearing procedure.

(a) Small and large intestinal tissues were harvested, treated with increasing concentrations of aqueous fructose solutions, and equilibrated in saturated fructose solution. The detailed protocol is described in Methods. (b) Transmittance during optical clearing. Gray values relative to the small intestine sample at 0 h were calculated by subtracting the background color under the tissues. Data are mean ± standard error of the mean (n = 5 each). (c) Transmission images were acquired using a Leica M205 fluorescence stereomicroscope under bright light (left) or UV (right). As a control, fluorescent dye (arrows) was dropped on the backing sheet. Intrinsic fluorescence was not detected.

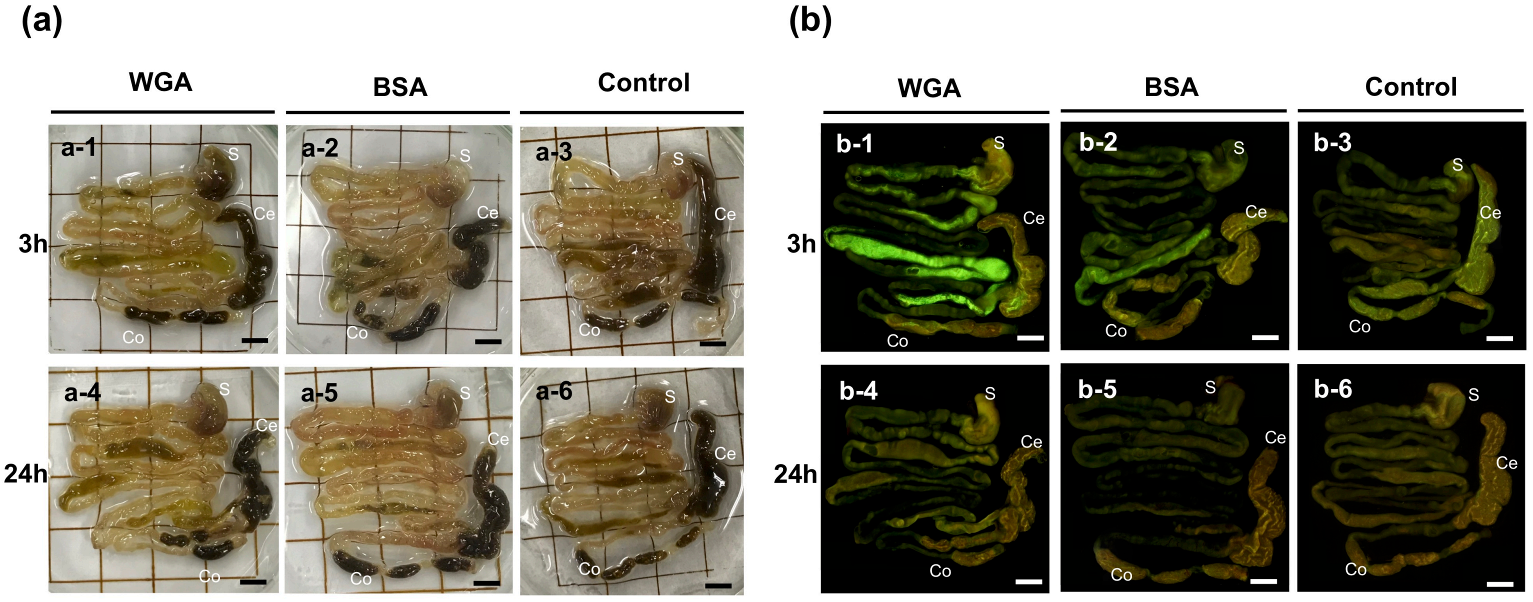

Figure S3. Visualization of the localization of wheat germ agglutinin (WGA)- or BSA-functionalized fluorescent microbeads in mouse whole GI tract using the tissue clearing method. WGA-beads (a-1, a-4, b-1, and b-4), BSA-beads (a-2, a-5, b-2, and b-5), or non-coated control beads (a-3, a-6, b-3, and b-6) were orally administered to mice. At 3 and 24 h after administration, whole GI tissues were treated with increasing concentrations of aqueous fructose solutions for tissue clearing. Whole-gut images were acquired using a Leica M205 fluorescence stereomicroscope under (a) bright light or (b) fluorescence. All data are representative of two independent experiments. Identical symbols indicate the same mouse tissue. Scale bar, 5 mm. S, stomach; Ce, cecum; Co, colon.

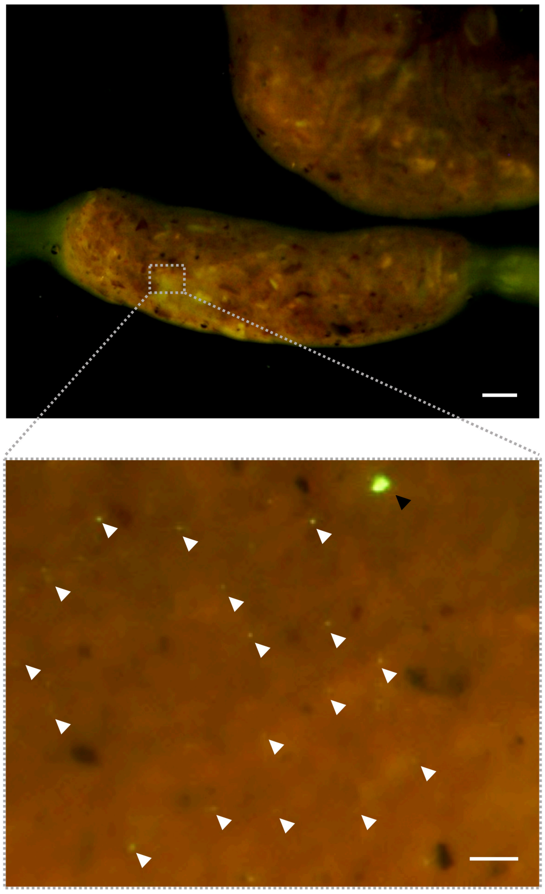

Figure S4. Micro-imaging of colon tissues.

Localization of EF-Tu-beads on the mucosal surface. Arrows indicate EF-Tu-beads. Black arrow indicates aggregation of EF-Tu-beads. Transmission images were acquired using a Leica M205 fluorescence stereomicroscope. Scale bar, 1.0 mm (upper panel) and 200 µm (lower panel).

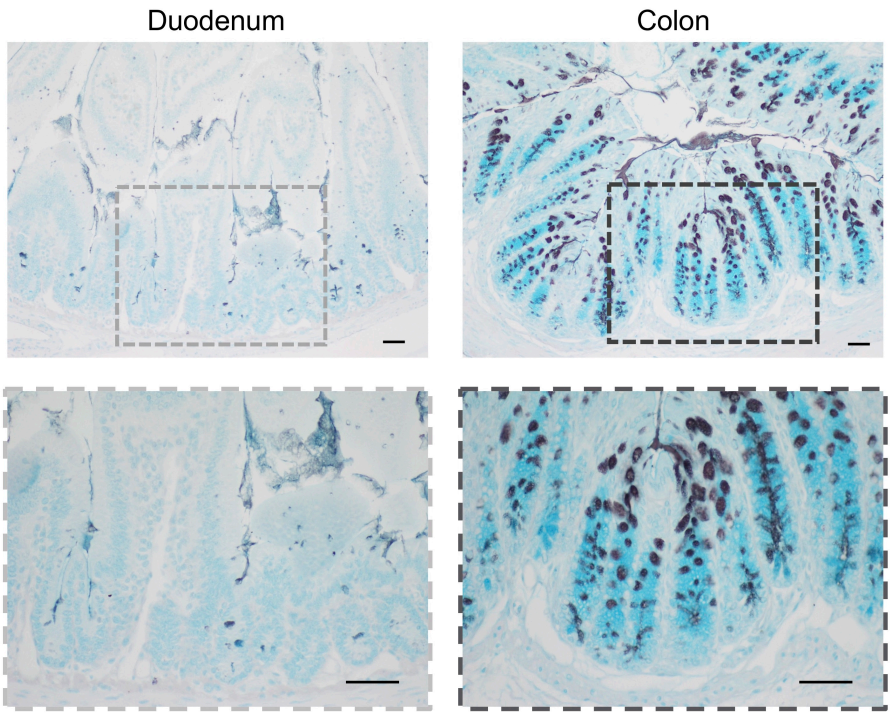

Figure S5. Histochemical staining of mouse mucosal surface with high iron diamine-alcian blue stain.

Histological analysis of duodenum (upper digestive tract) or colon (lower digestive tract) of C57BL/6J mice by means of high iron diamine-alcian blue staining (pH 2.5) histology. High iron diamine stain-positive area (dark brown) and alcian blue stain-positive area (blue) indicate sulfated and sialylated glycoconjugates, respectively. Scale bar, 50 μm.

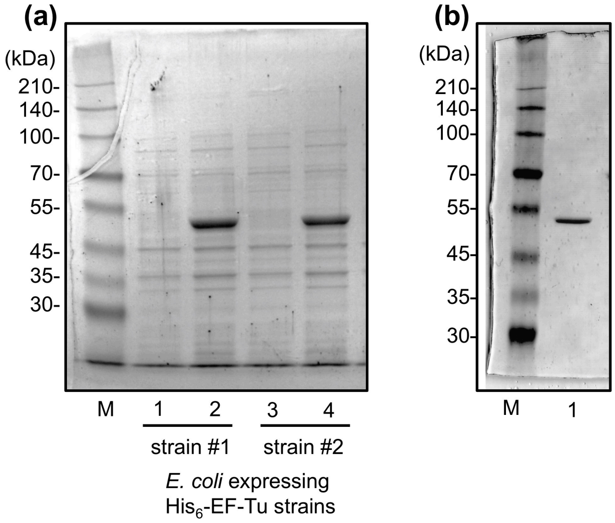

Figure S6. Expression and purification of EF-Tu protein from *L. reuteri* JCM1112^{T}.

(a) Whole cell lysates from *E. coli* expressing His$_6$-EF-Tu #1 and #2 strains before (lanes 1, 3) or after (lanes 2, 4) addition of IPTG were separated by SDS-PAGE and stained with Coomassie Blue. (b) The nickel-purified fraction (lane 1) derived from *E. coli* expressing His$_6$-EF-Tu #2 was resolved by SDS-PAGE and stained with Coomassie blue. M: molecular weight marker (Prestained XL-Ladder Broad, APRO SCIENCE, Tokushima, Japan). Masses are indicated in kDa to the left of the gel.
